# Editorial: Organoids as advanced model in cancer biology: the drug screening and clinical applications

**DOI:** 10.3389/fcell.2025.1700658

**Published:** 2025-10-28

**Authors:** Xiaoyong Dai

**Affiliations:** Department of Physiology, School of Medicine, State Key Laboratory of Bioactive Molecules and Druggability Assessment, and Guangdong Province Key Laboratory of Pharmacodynamic Constituents of TCM and New Drugs Research, Jinan University, Guangzhou, China

**Keywords:** cancer organoids, tumor precision medicine, drug screening, cancer immunotherapy, regenerative medicine

The field of cancer research has long been driven by the pursuit of more physiologically relevant and predictive model systems. Traditional two-dimensional cell cultures and animal models, while invaluable, often fall short of recapitulating the complex heterogeneity, spatial architecture, and dynamic microenvironment of human tumors. This gap has propelled the emergence of three-dimensional organoid cultures as a transformative technology. This Research Topic, “*Organoids as advanced model in cancer biology: the drug screening and clinical applications*,” was conceived to explore the rapidly evolving role of cancer organoids in refining drug discovery pipelines and bridging the daunting gap between preclinical findings and clinical utility. The articles published within this Research Topic collectively underscore the significant potential of organoid technology while also acknowledging the challenges that remain on the path to its widespread standardization and application.

This Research Topic significantly advances cancer organoid research by demonstrating their critical role in elucidating resistance mechanisms and enhancing drug screening. The study Agostini et al. “*Autophagy inhibition improves sensitivity to the multi-kinase inhibitor regorafenib in preclinical mouse colon tumoroids*” utilizes mouse colon organoids to reveal that autophagy inhibition overcomes regorafenib resistance by suppressing EMT and Erk1/2 activation, providing a novel combinatorial strategy for colorectal cancer. The other article Yehya et al. “*Repurposing piroxicam enhances the antineoplastic effects of docetaxel and enzalutamide in prostate cancer cells using 2D and 3D in vitro culture models*” employs prostate cancer organoids and 2D/3D models to show that repurposing piroxicam synergistically enhances the efficacy of conventional docetaxel and enzalutamide therapies, effectively reducing cell proliferation and organoid growth. The paper Varinelli et al. “*Organoids technology in cancer research: from basic applications to advanced ex vivo models*” reviews the broader utility of patient-derived organoids (PDOs), highlighting innovative techniques like decellularized ECM scaffolds to better recapitulate the tumor microenvironment (TME) for improved preclinical drug testing and personalized therapy prediction. Collectively, these contributions underscore organoids as physiologically relevant models that bridge basic research and clinical applications, offering powerful platforms for mechanistic discovery and therapeutic optimization in oncology.

In a broader context, this Research Topic illustrates a field in a state of exciting maturation. The published articles move beyond simply extolling the virtues of organoids and instead present critical data and analyses that probe their practical applications and limitations. The collective findings affirm that organoids serve as a crucial intermediate model, offering a much-needed human-relevant system that sits between conventional cell lines and patient trials. However, the journey is far from complete. Future directions must address the need to incorporate non-epithelial components—such as immune cells, fibroblasts, and vasculature—to create more complex tumor microenvironments that can be used to study immunotherapy and stromal interactions. Standardization of protocols for organoid generation, biobanking, and drug screening assays is also paramount to ensure reproducibility and allow for multi-institutional comparisons. Moreover, the ethical considerations surrounding the use of patient-derived tissues and the potential for these living biobanks to guide patient care require ongoing dialogue. In conclusion, the work presented in this Topic solidifies the standing of organoids as an advanced and indispensable model in cancer biology, poised to make significant contributions to drug development and the realization of personalized cancer medicine.

